# Pharmacological Inhibition and Genetic Knockdown of BCL9 Modulate the Cellular Landscape of Cancer-Associated Fibroblasts in the Tumor-Immune Microenvironment of Colorectal Cancer

**DOI:** 10.3389/fonc.2021.603556

**Published:** 2021-05-05

**Authors:** Mengxuan Yang, Zhuang Wei, Mei Feng, Yuanyuan Zhu, Yong Chen, Di Zhu

**Affiliations:** ^1^ Central Hospital of Minhang District, Shanghai, China; ^2^ Key Laboratory of Systems Biology, Innovation Center for Cell Signaling Network, CAS Center for Excellence in Molecular Cell Science, Institute of Biochemistry and Cell Biology, Shanghai Institutes for Biological Sciences, Shanghai, China; ^3^ School of Pharmacy, Fudan University, Shanghai, China; ^4^ Department of General Surgery, Huai’an Second People’s Hospital and the Affiliated Huai’an Hospital of Xuzhou Medical University, Huai'an, China; ^5^ New Drug Evaluation Center, Shandong Academy of Pharmaceutical Science, Jinan, China

**Keywords:** colorectal cancer, cancer-associated fibroblasts, Wnt signaling, BCL9, tumor immune microenvironment

## Abstract

Cancer-associated fibroblasts (CAFs) exert a key role in cancer progression and liver metastasis. They are activated in the tumor microenvironment (TME), but their prometastatic mechanisms are not defined. CAFs are abundant in colorectal cancer (CRC). However, it is not clear whether they are raised from local tissue-resident fibroblasts or pericryptal fibroblasts and distant fibroblast precursors, and whether they may stimulate metastasis-promoting communication. B-cell lymphoma 9/B-cell lymphoma 9-like (BCL9/BCL9L) is the key transcription cofactor of β-catenin. We studied the TME of CRC with single-cell sequencing and consequently found that *Bcl9* depletion caused a pro-tumor effect of CAFs, while inhibition of abnormal activation of Wnt/β-catenin signal through *Bcl9* depletion benefited T-cell–mediated antitumor immune responses. We also identified and evaluated four types of CAFs in CRC with liver metastasis. In summary, we demonstrate cell type landscape and transcription difference upon BCL9 suppression in CAFs, as well as how CAF affects cancer associated immune surveillance by inhibition of Wnt signaling. Targeting the Wnt signaling pathway *via* modulating CAF may be a potential therapeutic approach.

## Introduction

Colorectal cancer (CRC) is a widespread malignancy and the third leading cause of cancer mortality worldwide (1). Most CRCs occur sporadically in patients without family history of intestinal diseases (2). An unhealthy diet rich in meat but poor in fruit and vegetables increases the rising incidence of CRC (3). Traditional treatment (i.e., surgery, radiotherapy, and chemotherapy combined with targeted drugs) has improved the treatment of CRC. For example, combinations of targeted drugs containing epidermal growth factor receptor (EGFR) antibody and vascular endothelial growth factor (VEGF) antibody with chemotherapy such as FOLFIRI, XELOX/CAPOX, or FOLFOX have been shown to prolong survival in patients with CRC (4). However, treatment of patients with advanced recurrent or metastatic CRC is still inadequate. Therefore, it is necessary to determine molecules and signaling pathways that are critical for CRC and to find new therapeutic targets.

The Wnt signaling pathway, a tightly regulated, receptor-mediated transduction pathway, is important to the embryonic development and adult tissue homeostasis. Aberrantly activated in many tumors and others diseases, Wnt signaling plays a significant role in CRC given that 80% of patient samples in a large-scale study displayed APC and β-catenin mutations (5). Thus, Wnt pathway has emerged as a prospective anti-cancer target in CRC.

CAFs are the predominant cellular components of the stroma relative to primary and metastatic CRC (6, 7). Cancer cells can recruit CAFs to the TME by upregulating the expression of tissue inhibitors of metalloproteinases 1 (TIMP-1), which leads to increased CAF proliferation and migration by binding with CD63, its receptor on CAFs. The stroma of human CRCs expresses higher levels of TIMP-1 than its phenotypically normal counterpart (8).

CAFs can promote CRC cell proliferation, restrain tumor cell death, and elude tumor growth suppressors (9). CAFs of colorectum can discharge multiple growth factors, such as EGF, HGF, IGF1/2, PGE-2, PDGF, VEGF, and FGF-1 (10–12). For example, IGF1 can crosstalk with insulin-like growth factor 1 receptor (IGF-1R), thereby activating the mitogen-activated protein kinase (MAPK) and phosphoinositide 3-kinase (PI3K) signal pathways, and promoting cancer cell growth and survival (13). Moreover, HGF secreted by CAFs can phosphorylate the receptor tyrosine kinase c-Met and human epidermal growth factor receptor (EGFR2/EGFR3) in CRCs, and activate both MAPK and PI3K/AKT pathways (14). Consequently, these growth factors released by CAFs induce tumor cells proliferation and tumorigenicity in CRCs (15). Moreover, Hawinkels et al. demonstrated that the interaction between CRC cells and resident fibroblasts results in TGF-β1 signaling hyperactivation and differentiation of the resident fibroblasts into α-SMA^+^ CAFs, which conversely leads to synthesis of TGF-β and proteinases in the TME, thus generating a cancer-promoting feedback cycle (10).

Tumor metastasis refers to a multistage process in which malignant cancer cells migrate to the surrounding tissues and continue their proliferation from the primary site through lymphatic channels, blood vessels, or body cavities. In this process, tumor cells detached from the primary site violate other tissues and eventually lead to metastatic tumors. Malignant tumor metastases are the main reason for the failure of tumor treatment. CAFs are abundant in CRC and play a critical role in cancer progression. They are enriched in the TME, but their prometastatic mechanisms in CRC with metastasis have not been defined (9, 10).

First, chemokines in the TME are conducive to tumor metastasis. In CRCs, PDGF-activated CAFs can facilitate tumor cells intravasation and distant metastases by secreting stanniocalcin1 (STC1) (16). Second, through increasing the production of extracellular matrix (ECM) and proteolytic enzymes, stromal TGFβ signaling promotes tumor metastasis (17, 18). It was shown that CAFs can lead to TGFβ signaling hyperactivation after incubation with supernatants from CRC cells (18). Moreover, CAFs are the main source of connective tissue constituents of the TME, including collagens and proteoglycans (19, 20). Third, the expression of tumor-promoting MMPs is higher in CAFs than in CRC cells (18, 20). Type IV collagen, a base for MMP-2 and MMP-9, is one of the key components of the basement membrane, which is essential for tumor invasion and metastasis (18, 21). Cancer cells may undergo epithelial mesenchymal transition (EMT), a transdifferentiation programme to get a highly invasive phenotype (22). Snail1 is an important transcriptional factor that exerts critical role in EMT. Snail1 expression is higher in colon CAFs than in normal fibroblasts, and it correlates with CRC cells migration and proliferation (23). Further studies revealed that a mechanically active heterotypic E-cadherin/N-cadherin adhesion between the CAFs and tumor cells can enable fibroblasts to drive cancer cell invasion (24).

Even though CAFs have been reported to be involved in tumorigenesis, angiogenesis, and metastasis, CAF-related anti-tumor immune response in CRC is still not clear. Apart from potent protumorigenic ability, some CAF subsets have tumor-inhibiting functions, which further supports the concept of CAF heterogeneity. Interestingly, there are at least two CAF subsets in the breast TME that can be distinguished by CD146 expression (25). Specifically, CD146^+^ CAFs can lead to persistent estrogen-dependent proliferation and breast cancer cells sensitivity to tamoxifen, while CD146^−^ CAFs can suppress estrogen receptor expression and response of cancer cells to estrogen, thereby resulting in tamoxifen resistance (25). Additionally, CAF peripheral cells in CRC show upregulation of podoplanin, a mucin-type transmembrane glycoprotein. Podoplanin is a significant indicator of favorable prognosis in patients with advanced CRC as shown in multivariate analysis of both disease-free survival and liver metastasis-free survival (26). Apart from this, upon co-culturing with CAFs treated with siRNA for podoplanin, CRC cells demonstrated decreased cell invasion, suggesting a protective role of CAFs against CRC cell invasion (26).

## Materials and Methods

### Compounds and Short Hairpin RNA

hsBCL9_CT_-24 was purchased from AnaSpec (Fremont, CA, USA) based on previous protocols (27). Analytical high-performance liquid chromatography (HPLC) and mass spectrometry (MS) were used to evaluate the synthesis and purification of peptides. hsBCL9_CT_-24 was dissolved as a 10 mmol/L solution and was diluted prior to assay. pGIPZ- and/or pTRIPZ (inducible with doxycycline)-based lentiviral shRNAs for mouse *Bcl9* shRNA#5 (V3LMM_429161), mouse *Bcl9l* shRNA#1 (V2LMM_69221), and non-targeting shRNA were obtained from Open Biosystems/GE Dharmacon. The non-targeting (NT) lentiviral shRNA expressing an shRNA sequence with no substantial homology to any mammalian transcript was used as a negative control.

### Animal Tumor Specimens

For each tumor, at least four regions were sampled, including two regions in inner layers and two regions in outer layer. In total, six samples from six mice were collected. Detailed information is summarized in **Supplementary Table 1**. All the procedures were performed in accordance with the protocols approved by the School of Pharmacy in Fudan University’s animal care committee (Approval number: 2020-04-YL-ZD-02) and with the guidelines of the Association for Assessment and Accreditation of Laboratory Animal Care International.

### Specimen Processing

Fresh tumors from mice were collected in MACS Tissues storage solution (130-100-008, Miltenyi Biotec, Germany) in the operation room after surgical resection, and immediately transferred to Fudan laboratory for processing. Tissues were minced into pieces < 1 mm^3^ on ice, shifted to a C tube (130-093-237, Miltenyi Biotec), and enzymatically digested by MACS Tumor Dissociation Kit (130-095-929, Mitenyi Biotec) in line with corresponding programs. The resulting suspension was filtered through a 40-μm cell strainer (Falcon) and washed by RPMI 1640 (C11875500BT, Gibco). Erythrocytes were removed by adding 2 ml Red Cell Lysis Buffer (555899, BD Biosciences). A Dead Cell Removal Kit (130-090-101, Miltenyi Biotec) was subsequently used to enrich live cells. After resuspension in RPMI 1640 (C11875500BT, Gibco), single-cell suspension was obtained. Trypan blue (15250061, Gibco) was next used to check whether cell viability was >90% to be qualified enough for library construction.

### 10× Library Preparation and Sequencing

Cell concentration was adjusted to 700–1200 cells/µl to run on a Chromium Single-Cell Platform (10× Genomics Chromium™). 10× library was generated in accordance with the manufacturer’s protocol of 10× genomics Single Cell 3′ Reagent Kits v2. The clustering was carried on a cBot Cluster Generation System with TruSeq PE Cluster Kit v3. Qubit was used for library quantification. The final library was sequenced on an Illumina HiSeq3000 instrument using 150 bp paired-end reads.

### Principal Component Analysis and t-SNE Analyses

The total number of unique molecular identifiers (UMIs) per cell was counted for the number of UMI sequences of high-quality single cells and genes in the sample. To normalize the number of UMIs in each cell to the median of the total UMI of all cells, we used the median normalization process. The similarity between cells was investigated by means of Principal component analysis (PCA) reduction dimension. The closer the expression trend of cellular genes, the closer the sample distance was. Using t-distributed stochastic neighbor embedding (t-SNE) to visualize the single cell clustering for the top 10 principal components of the PCA resulted in the largest variance explained. The protocol of the t-SNE presentation method was to recount the sample distance through the conditional probability of random neighbor fitting according to the Student’s *t* distribution in the high dimensional space, so that the sample presented a clearly separated cluster in the low dimensional space.

### Pathway and Functional Annotation Analysis

Through DAVID (https://david.ncifcrf.gov/), we conducted Kyoto Encyclopedia of Genes and Genomes (KEGG) pathway annotation and enrichment. KEGG is a database resource for investigation of the high-level functions and effects of the biological system (http://www.genome.jp/kegg/). Pathways with a Q value ≤0.05 were defined as significantly enriched. Analysis was performed base on The Gene Ontology database conducted the functional annotation, including biological process, cellular component, and molecular function classifications. To select only significant categories, we used a Fisher’s exact test, and the GO terms with computed Q value ≤0.05 were considered as significant.

### Gene Prognostic Performance in TCGA Samples

We downloaded the TCGA datasets, including COAD and READ, from cBioPortal (http://www.cbioportal.org/). Based on gene median expression level, the CRC samples were divided into the high- and low-expression groups. To compare the overall and disease-free survival among two groups, Kaplan-Meier curve was constructed. Log-rank P value and hazard ratios (HR) were calculated with SPSS 22.0. In TIMER analysis, correlation analysis of gene expression in tumor-infiltrating immune cells was performed.

### Single Cell Data of Human Tumor Sample

Samples from patients with CRC were analyzed from the data base (SUB8333842). The study was approved by the Ethics Committee of Fudan University. Informed consent was obtained from every patient who agreed to provide specimens for scientific research.

### Cell-Cell Interaction Analysis

Based on the above analysis of the expression abundance of ligand–receptor, we obtained the number of ligand–receptor interactions between two cell types, allowing a preliminary assessment of the communication relationship between the cells. To identify biological relevance, we used cellPhoneDB software to perform pairwise comparisons between all cell types in the dataset, and analyze the number of significantly enriched ligand–receptor interactions between two cell types. First, we randomly permuted the cluster labels of all cells (1,000 times by default) and determined the mean of the average ligand expression level in a cluster and the average receptor expression level in the interacting cluster. In this way we generated a null distribution for each ligand–receptor pair in each pairwise comparison between two cell types. We obtained a *P* value for the likelihood of cell-type enrichment of each ligand–receptor pair. Wilcoxon rank sum test was used for statistical analyses.

### GSVA Analysis

For CAFs and tumor cell population expression data, the average expression of each gene of the corresponding cell was calculated in each sample. GSVA analysis was performed on the sample expression data of the above four types of cells using the C2 KEGG pathway subclass data in the MsigDB database to obtain the GSVA score of each pathway in each sample. For the above four types of cell sample expression data, the C2 KEGG pathway subtype data were used in the MsigDB database to perform GSVA calculation Z-score analysis to obtain the GSVA Z-score of each pathway in each sample. The limma package in R was used to calculate the GSVA score data of the above four types of cells. Metastatic vs. nonmetastatic grouping was applied to calculate the difference, and obtain the *t* score and *P* value of each KEGG pathway. The pathways were filtered according to the marked P value and a bar chart was prepared based on *t* score. Corresponding Z-score was extracted for heatmaps based on selected channels.

### Statistical Analysis

SPSS 22.0 was used in data analysis, and *t* test was used to determine the statistical significance. P values <0.05 were considered statistically significant.

## Results

### Single-Cell RNA-seq of Mouse CT26 Tumor With Pharmacological Inhibition of BCL9

**Figure 1:**
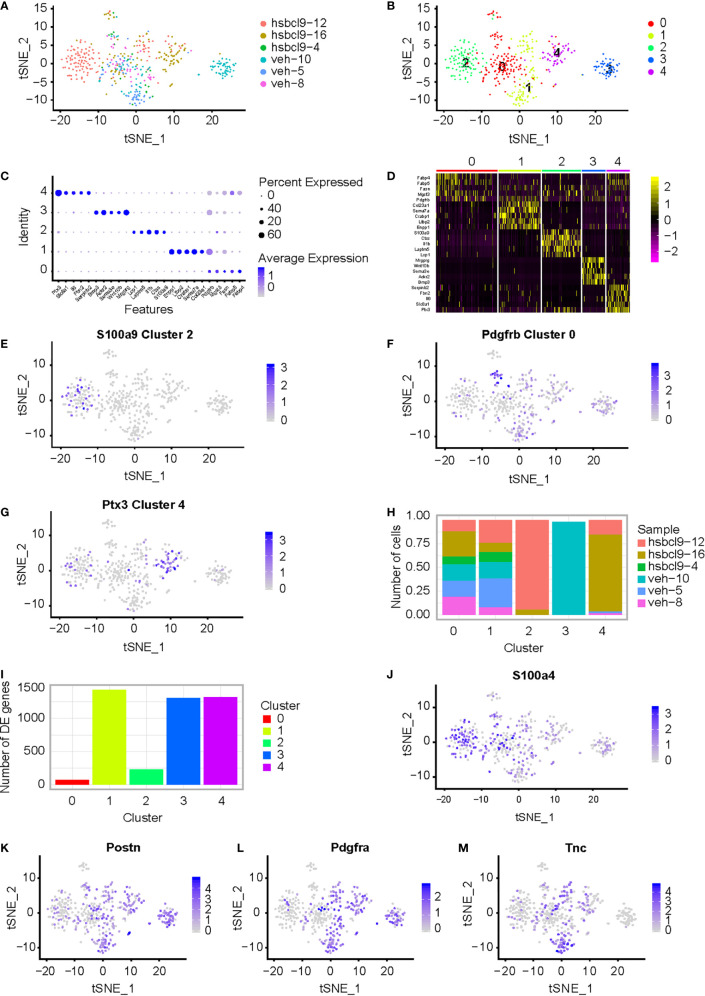
CAF analysis in mouse CT26 tumor in hsBCL9_CT_-24 treatment. **(A)** Single cell RNA-seq of mouse CT26 tumor; t-SNE of the six samples from CT26 tumor mice, which were treated by hsBCL9_CT_-24 or by vehicle, were collected. **(B)** t-SNE plot expression of marker genes for the five different markers of tumor cell defined above each panel color-coded by their associated cluster. Five clusters high quality cells were collected for further analyses. **(C)** Bubble plots express different marker genes according to each cluster high specific expression gene separate different cells. **(D)** Five cluster cells were analyzed for different genes with heatmap. **(E–G)** Expression of marker genes for the cell types defined above each cluster; *S100a9*, *Pdgfrb*, and *Ptx3*, highly expressed in clusters 2, 0, and 4, respectively. **(H, I)** Different clusters present difference of cell numbers and genes among clusters 0, 1, 2, 3, and 4. **(J–M)** Expression of CAF marker genes for the cell types defined above each cluster; *S100a4*, *Postn*, *Pdgfra*, and *Tnc*, highly expressed in clusters 2, 0, and 4, respectively. Wilcoxon rank sum test was used for statistical analyses.

Cancer-associated fibroblasts (CAFs) are very critical in cancer progression when they are activated in the TME. To determine and specify whether they exhibit prometastatic mechanisms, we characterized the heterogeneity of mouse CT26 tumor in hsBCL9_CT_-24 treatment. Based on our previous study (28), we conducted structure–activity relationship analyses in SAH-BCL9*_B_* to generate the newly generated peptide series, among which hsBCL9_CT_-24 was found to potently disrupt Bcl9/β-catenin and demonstrate the most potent *in vitro* activity (29). Cancer-associated fibroblasts were investigated in these tumors. Six samples from six CT26 mice with a tumor were collected, and they were treated by hsBCL9_CT_-24 or by vehicle as control. Tissues were minced rapidly and enzymatically digested with MACS Tumor Dissociation Kit according to corresponding procedure to a single-cell suspension, and run on a Chromium Single-Cell Platform (see methods). 10× library was generated according to the manufacturer’s protocol. The clustering was implemented on a cBot Cluster Generation System with TruSeq PE Cluster Kit v3. Qubit was used for library quantification. The final library was sequenced on an Illumina HiSeq3000 instrument. Results of scRNA-seq with unique transcript counting through barcoding with UMIs (see Methods) were analyzed. Using t-SNE to group cells with similar expression profiles, graph-based clustering was run to build a sparse nearest-neighbor graph without prespecification of the number of clusters (**Supplementary Figure 1A** and **Figure 1A**). We identified five clusters (**Figure 1B**) based on the t-SNE dimensionality reduction and unsupervised cell clustering. The mean expression of every gene was calculated across all cells in the cluster, and the log2 fold change of differentially expressed genes was counted relative to the other clusters identified relevant genes that were enriched in different clusters. High expression of significant genes with the known markers of major cell types is shown in **Figure 1C**. The relevant genes were enriched by heatmaps of the clusters 0, 1, 2, 3, and 4. Five transcription factors showed the highest difference (log2 fold change) in expression regulation estimates between different clusters (**Figure 1D**). Each cluster was defined by expression of marker genes for the cell types (**Figures 1E–G**); *S100a9*, *Pdgfrb*, and *Ptx3*, were highly expressed in clusters 2, 0, and 4, respectively. Different clusters present difference of cell numbers and difference of genes in cluster 0, 1, 2, 3, and 4 (**Figures 1H, I**). Cluster 2 with high expression of *S1009a* was defined as CAF4. t-SNE of additional CAF marker genes, such as *S100a4*, *Pdgfa*, *Postn*, *Tnc*, was shown. (**Figures 1J–M**). *Bcl9* expression in different clusters in vehicle and hsBCL9_CT_-24 group was also determined by t-SNE and UMAP (**Supplementary Figures 1C, E** **)**.

### Single-Cell RNA-seq of Mouse CT26 Tumor With Genetic Knockdown of *Bcl9*

**Figure 2:**
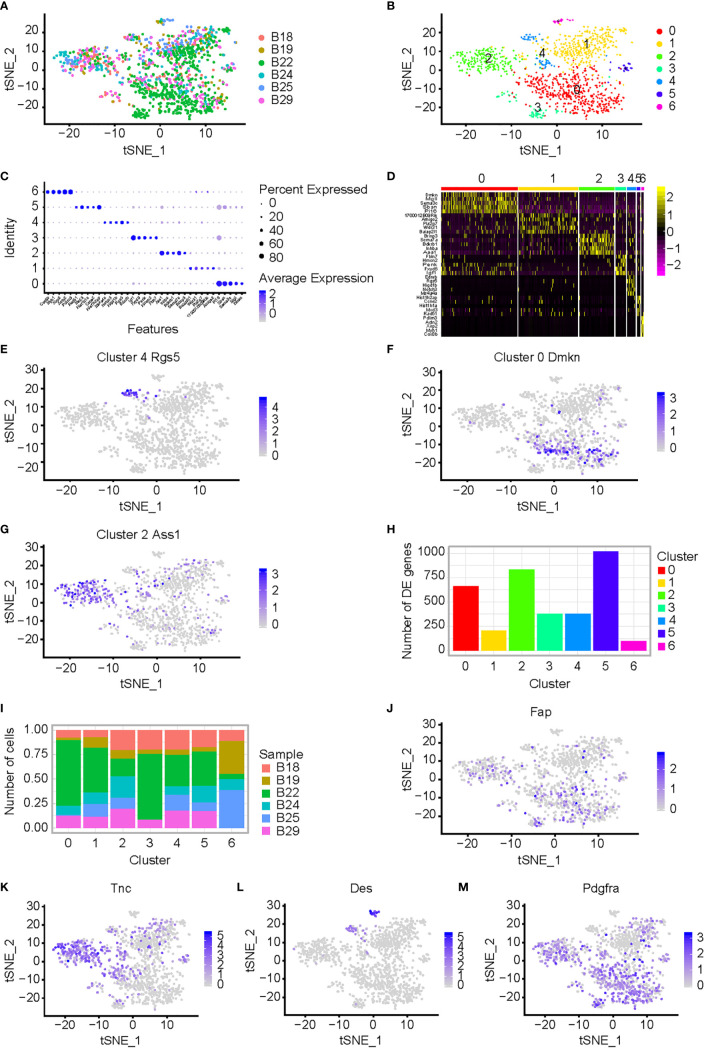
CAF analysis in mouse CT26 tumor with BCL9 depletion. **(A)** Single cell RNA-seq of CT26 tumor cells; t-SNE of the six samples from CT26 tumor cells, which were treated by shRNA-BCL9 or NT-shRNA, were collected. The plasmid with shRNA-BCL9 was constructed and transfected into CT26 cells. The CT26 cells which transfected NT-shRNA were used as control. Six samples included: B18 (NT-shRNA sample 18), B19 (NT-shRNA sample 19), B22 (NT-shRNA sample 22), B24 (BCL9-shRNA sample 24), B25 (BCL9-shRNA sample 25), and B29 (BCL9-shRNA sample 29). **(B)** Seven distinct cell clusters, named as C0–C6 based upon their total cell numbers, expressed unique transcriptional profiles and marker gene. **(C)** Bubble plots show significant genes with high expression of the known markers of major cell types. **(D)** Heatmap shows significant difference in gene expression among seven clusters. **(E–G)** Expression of marker genes for the cell types defined above each cluster; *Rgs5*, *Dmkn*, and *Ass1*, highly expressed in clusters 4, 0, and 2, respectively. **(H, I)** Different clusters present difference in cell numbers and genes between clusters 0, 1, 2, 3, 4, 5, and 6. **(J–M)** Expression of CAF marker genes for the cell types defined above each cluster; *Fap*, *Tnc*, *Des*, and *Pdgfra*, highly expressed in clusters 2, 0, and 4, respectively. Wilcoxon rank sum test was used for statistical analyses.

The plasmid with knockdown (KD) shRNA-BCL9 was constructed and transfected into CT26 cells (**Supplementary Figure 1B**). The CT26 cells transfected with non-targeting (NT) shRNA were used as controls. CT26 expressing NT-shRNA and shRNA-BCL9 cells were implanted subcutaneously in Balb/c mice. Six tumor samples were operated (B18, NT-shRNA sample 18; B19, NT-shRNA sample 19; B22, NT-shRNA sample 22; B24, BCL9-shRNA sample 24; B25, BCL9-shRNA sample 25; and B29, BCL9-shRNA sample 29). Graph-based clustering was handled using t-SNE to gather cells with similar expression profiles and to construct a sparse nearest-neighbor graph without prespecification of the number of clusters (**Figure 2A** and **Supplementary Figure 1A**). We defined seven different cell clusters, named as C0–C6 according to their total cell numbers, presenting unique transcriptional profiles (**Figure 2B**).

We identified genes enriched in a different cluster through high expression of the known markers of major cell types shown in **Figure 2C**. We identified seven clusters (0, 1, 2, 3, 4, 5, and 6). The relevant genes were enriched by heatmap of the clusters 0, 1, 2, 3, 4, 5 and 6 (**Figure 2D**). Marker genes of the cell types defined above each cluster, *Rgs5, Dmkn*, and *Ass1*, were highly expressed in clusters 4, 0, and 2, respectively (**Figures 2E–G**). Different clusters presented different genes and different cell numbers (**Figures 2H, I** **)**. Cluster 4 with high expression of *Rgs5* was defined as CAF4. t-SNE of additional CAF marker genes, such as *Fap*, *Tnc*, *Des*, *Pdgfra*, was shown. (**Figures 2J–M**). *Bcl9* expression in different clusters in NT-shRNA and Bcl9-shRNA group was also performed in t-SNE and UMAP (**Supplementary Figures 1D, F** **)**.

### Signaling Pathway and Transcription Factor Analysis

**Figure 3:**
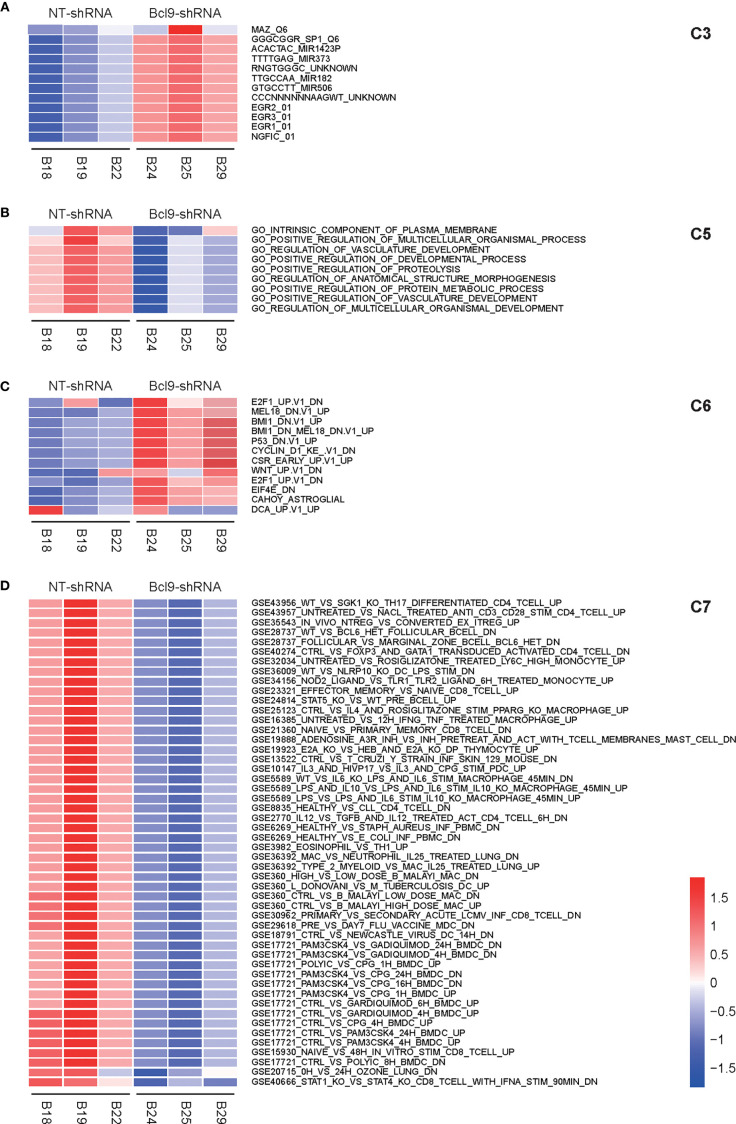
GSVA analysis of CAF in mouse CT26 tumor. **(A)** Motif gene sets a direct comparison of tumor cells between NT group and Bcl9 knockdown group, revealing IK3 and IRF pathways as the enriched signatures in tumor cells. **(B)** Gene ontology relative regulation of vasculature development gene is the enriched signature in NT tumor cells. **(C)** Oncogenic signatures manifest PDG, MTOR, and PETEN pathways as the enriched signatures in NT tumor cells. **(D)** Immunologic signatures pathway, such as TGF, is the enriched signature in tumor cells.

GSVA analysis was performed on the sample expression data of the above four types of cells (NT-shRNA and BCL9-shRNA) using the C2 KEGG pathway subclass data in the MsigDB database to obtain the GSVA score of each pathway in each sample. For the above four types of cell sample expression data, the C2 KEGG pathway subtype data were used in the MsigDB database to obtain the GSVA Z-score of each pathway in each sample. NT tumor vs knockdown group was applied to calculate the difference, and obtain the *t* score and P value of each KEGG pathway. The pathways were filtered based on the marked P value, and a bar chart was created based on *t* score. Corresponding Z-score was extracted for heatmap based on selected channels. Motif gene sets a direct comparison of tumor cells between NT group and *Bcl9* knockdown group revealed IK3, IRF pathway as the enriched signature in tumor cells (**Figure 3A**). Gene ontology relative regulation of vasculature development gene was the enriched signature in NT tumor cells (**Figure 3B**). Oncogenic signatures manifested PDG, MTOR, and PETEN pathway as the enriched signature in NT tumor cells (**Figure 3C**). We further explored immunologic signatures pathway and revealed that immunologic gene such as IL-2 and STAT1 was enriched in tumor cells (**Figure 3D**).

**Figure 4:**
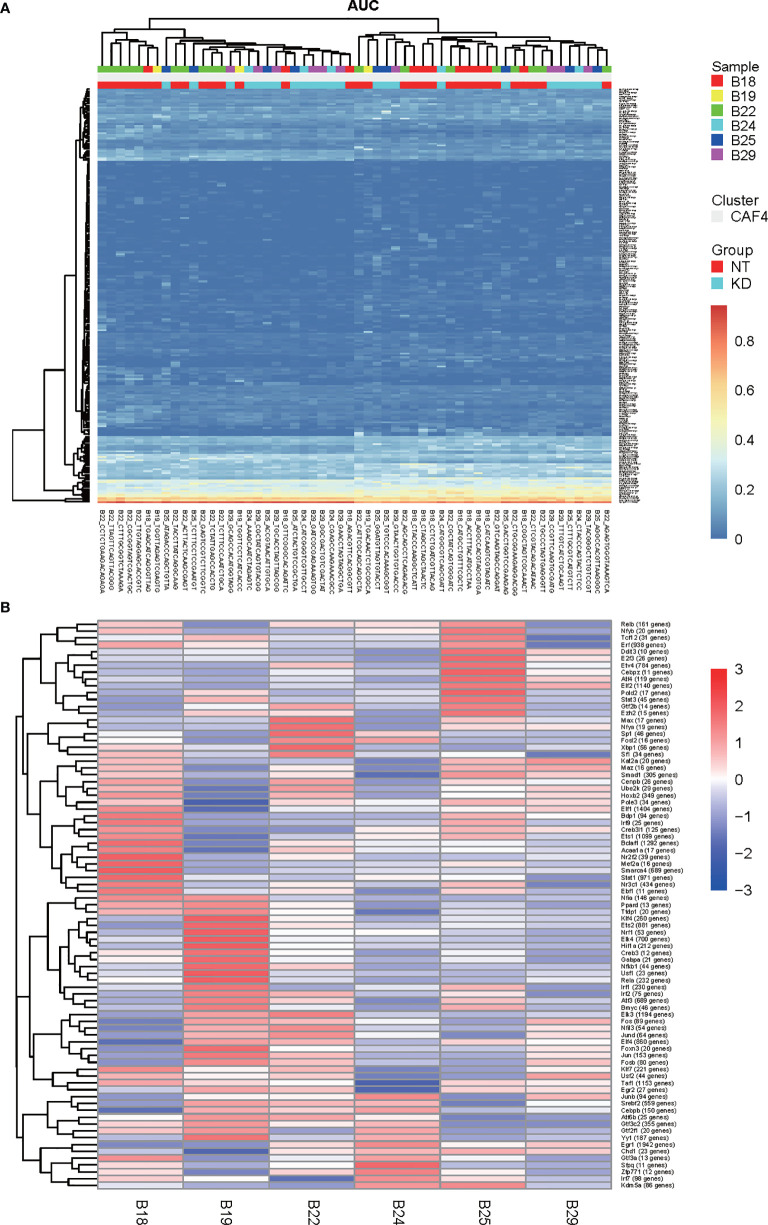
SCENIC analysis of CAF in mouse CT26 tumor **(A, B)**. Single cell regulatory network inference and clustering (SCENIC) analysis. By using SCENIC to predict transcription factors and corresponding target genes, a transcription factor regulatory network module was constructed. By calculating the area under the curve (AUC), the higher the AUC value, the stronger the regulatory activity of transcription factors to activate target gene expression **(A)** SCENIC analysis to assess transcription factors which underlie differences in expression of CT26 tumor cell between NT and *Bcl9* KD tumor cells. **(B)** Average activity of regulons in different cell subpopulations.

Single cell regulatory network inference and clustering (SCENIC) is a statistical method for simultaneous gene regulatory network reconstruction as well as cell-state identification from single cell RNA-seq data (http://scenic.aertslab.org). SCENIC can furnish unique insights into the mechanisms driving cellular heterogeneity. We applied SCENIC analysis to investigate the transcription factors underlying differences in CT26 tumor cell expression between NT and *Bcl9* KD tumor cells. We also identified Foxp4 as a candidate transcription factor underlying gene expression differences in NT and *Bcl9*-KD tumor cells (**Figures 4A, B**).

### Cells Cross Talk Between CAFs and CT26 Tumor Cells

Tumorigenesis and tumor cells proliferation and metastasis always depend on tumor stromal cells. The TME has diverse functions, including matrix deposition and reforming tumor cells, and significantly influences therapeutic response and clinical outcome, through the circulatory and lymphatic systems interplay with surrounding cells to influence the development and progression of cancer (30). In addition, stromal cells such CAFs in the TME play indispensable roles in all stages of carcinogenesis by stimulating and facilitating uncontrolled cell proliferation. CAFs’ extensive corresponding signaling interacts with tumor cells and cross talks with infiltrating leukocytes. In turn, tumor cells transform the TME to favor for tumor growth (31).

**Figure 5:**
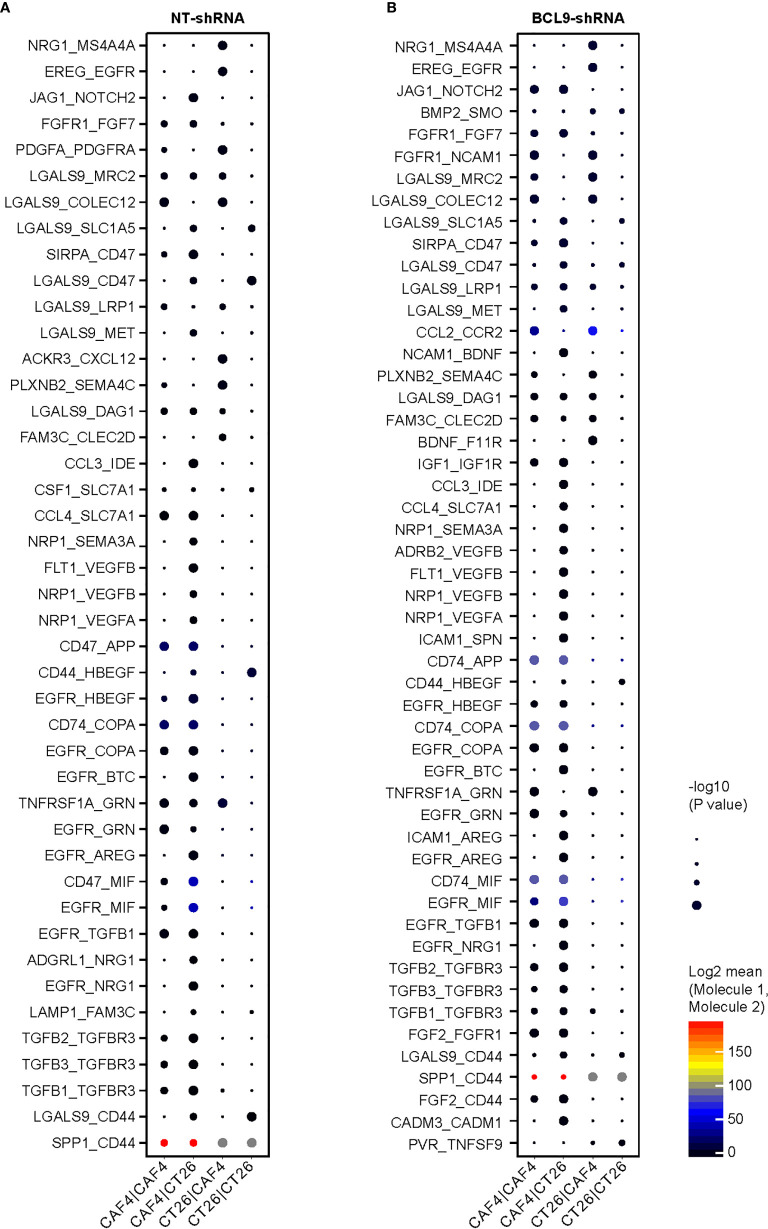
Tumor CAF interaction in mouse CT26 tumor. Cell interaction in normal tumor (NT) and knockdown (KD) groups. **(A)** The binding between receptor and ligand is obvious in CAF/CT26 in NT subgroups. **(B)** The binding between receptor and ligand is obvious in CAF/CT26 in KD subgroups.

Analysis of the number and expression of ligand receptor pairs in CAF cells and CT 26 cells pairs was based on single cell gene expression matrix by CellPhoneDB software and construction of cell interaction network graph, so as to predict the potential communication between cells. The interaction was determined by calculating the average expression of receptors and ligands. After calculating the score for each ligand and receptor, we averaged the interaction score of the tumor model to determine the conservative interaction. According to the above results, CAFs were shown as a potential target for optimizing therapeutic strategies against cancer. We explored the cell interaction between NT and *Bcl9*-KD groups. The TGFβ-TGFβR and EGFR-NRG1 binding between receptor and ligand was obvious in CAF/CT26 subgroups, revealing a cross-talk between the CAFs and CT26 tumor cells through TGF pathway, which affects the expression of EGFR to transform the tumor cells (**Figures 5A, B**).

### Heterogeneity of Human Cancer-Associated Fibroblasts

We used a database which included nine colon samples from colonic cancer patients, five of which were with metastasis and four without metastasis. We implemented principal component analysis on genes variably expressed across all 69,548 cells (n = 26,805 genes). We then sorted cells into types using graph-based clustering on the informative principal components (**Supplementary Figures 2A–C**). This technique identified cell clusters that, through marker genes, included cancer cells—markers *EpCAM, KRT8, KRT18*, and *KRT23*, fibroblasts—marker *COL1A1* (cancer cells, 4389 cells; fibroblasts, 2549 cells; **Supplementary Figures 2D, E**). We also compared difference of fibroblast enrichment between metastatic and nonmetastatic samples. We found that fibroblasts were increased in metastatic CRC compared with nonmetastatic CRC (**Supplementary Figure 2F**).

To compare with the findings obtained from mouse CAFs, we explored heterogeneity of human CAFs. In total, 2549 fibroblasts were detected among 9 samples. Next, we focused on CAFs in right-sided CRC-LM, and revealed seven subtypes in subclustering (**Supplementary Figure 2G**). A subset showing α-smooth muscle actin (α-SMA), fibroblast activation protein (FAP), periostin, neuron glial antigen-2 (NG2), tenascin-C, platelet-derived growth factor receptors α and β (PDGFR α and β), desmin, vimentin, and fibroblast specific protein-1 (FSP-1) was defined as CAFs. The cellular identity and origin of CAFs stem from various lineages, and the subpopulations we detected were highly consistent with the fibroblast identity. Analysis partitioned CAFs into four types (cluster 5, cluster 0, cluster 10, and cluster 14; **Supplementary Figure 2H**), where cluster 0 expressed *ESAM*, cluster 5 expressed *COL12A1*, cluster 14 expressed *POSTN*, and cluster 10 expressed *CFD* (**Supplementary Figure 2H**). We identified distinct populations of CAFs, including matrix CAFs (mCAFs) in cluster 5, vascular CAFs (vCAFs) in cluster 0, myofibroblastic CAFs (myCAFs) in cluster 14, and inflammatory CAFs (iCAF) in cluster 10 (**Supplementary Figure 2H**), which had high levels of cytokines and chemokines (32, 33).

Gene ontology annotation and enrichment were analyzed. In mCAFs, extracellular matrix organization, extracellular structure organization, and cell proliferation were significantly enriched (**Supplementary Figure 2I**). KEGG pathways analysis in CAF clusters was performed. For example, in CAF1, Wnt signaling and NF-κB signaling were enriched (**Supplementary Figure 2J**), indicating that CAFs are involved in complex structural and paracrine interactions in the TME, consistent with intratumoral CAF heterogeneity.

### Cluster and Survival Analysis of Human Colon Cancer

**Figure 6:**
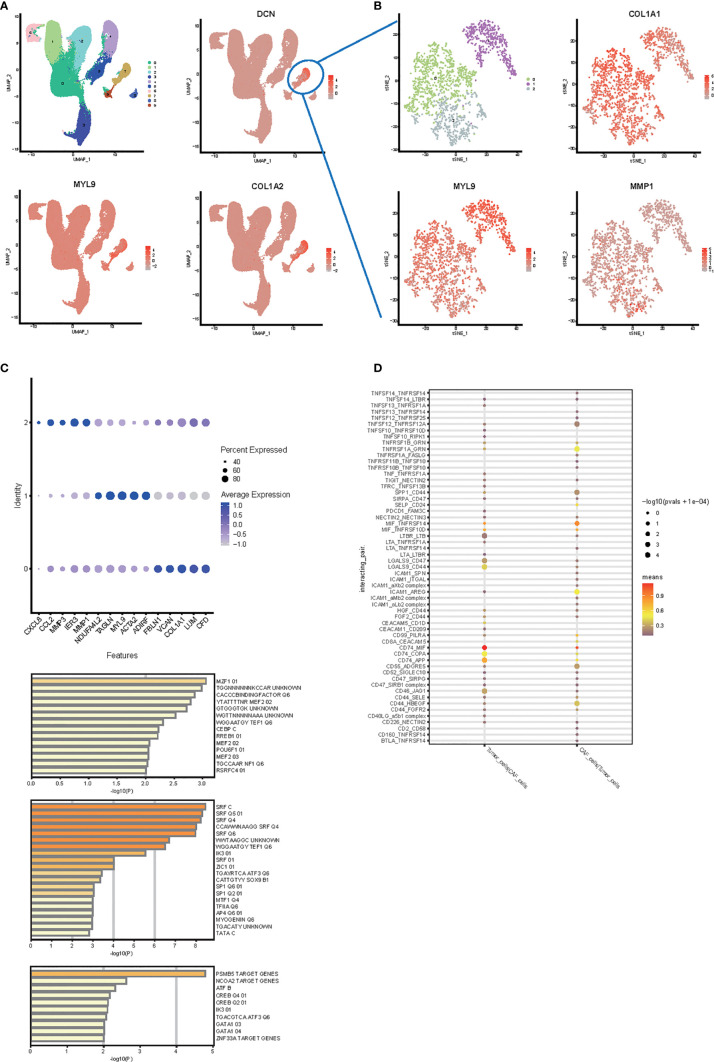
Single cell RNA-seq analysis in human colon cancer samples. **(A)** UMAP plot and clustering analysis. The color map showed clusters, and the markers of CAF were plotted on the map (*DCN, MYL9, COL1A2*). Cluster 2 was identified as cancer cells and cluster 7 was identified as CAF. **(B)** t-SNE plot and clustering analysis for CAF subtype. The color map showed clusters, and the markers of each CAF subpopulation were plotted on the map (*MMP1, MYL9, COL1A1*). **(C)** Bubble plots express different marker genes according to each cluster high specific expression gene separate different cells of CAF. **(D)** Transcription factor analysis from metascape. The first 50 genes of each CAF subpopulation were used to analyze the transcription factors specifically regulated. **(E)** Cell interaction between CAF and tumor cell.

To compare cell cluster and type profiles in tumor, we performed scRNA-seq in parallel in non-metastasis and metastasis CRC. We analyzed tumor samples from 13 colon cancer patients (SUB8333842). In the preliminary clustering (**Figure 6A**), we separated CAF cells and tumor cells. Extract the CAF cell population and perform re-clustering analysis (**Figure 6B**). As a result, the CAF cell subgroups can be divided into 3 clusters (**Figures 6B, C** **)**. The expression patterns of these three clusters are comparable to those of mice. They mainly expressed collagen production, muscle-like and stromal cells related markers, such as MMP1, MYL9, COL1A1, etc (**Figures 6B, C** **)**. In order to study which transcription factors are related to the formation of these subgroups, we conducted a Metascape analysis. As a result, shown in **Figure 6D**, the establishment of cluster0, cluster1, and cluster2 are mainly related to the transcriptional regulation of MZF1, SRF, and PSMB5, respectively.

**Figure 7:**
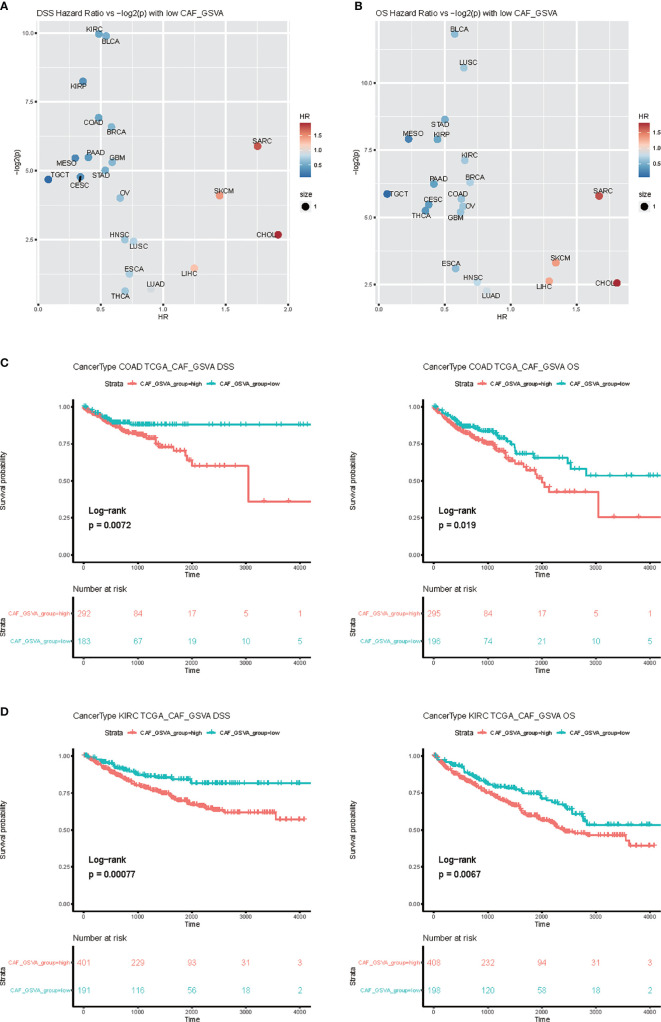
Survival analysis of CAF related genes. **(A)** Select each 50 genes (**Supplementary Table 1**) related to the patient’s CAF, and use GSVA to calculate the degree of enrichment in TCGA patients. Use this index to classify patients in different tumors and perform survival analysis. **(B)** The volcano graph of HR and p values, with Overall survival (OS) on the left and disease-specific survival (DSS) on the right. **(C, D)** Survival analysis of Kidney renal clear cell carcinoma (KIRC) and Colon Adenocarcinoma (COAD).

In order to study the impact of the key marker genes of the CAF cell subpopulation on the prognosis of tumor patients, we used the first 50 key marker genes in the three clusters indicated in **Figures 6B, C** to perform GSVA analysis, and took the GSVA value as the basis for classification, The survminer method was used for optimal grouping, and the two groups with high and low GSVA values were separated, and survival analysis was performed on the two groups. Taking the HR obtained by the survival analysis into a volcano map (**Figures 7A, B** **)**, it was found that the key marker genes of the CAF cell subpopulation had the greatest impact on the Kidney renal clear cell carcinoma (KIRC), and colon cancer Colon Adenocarcinoma (COAD) was also in the highly significant range (**Figures 7C, D** **)**.

## Discussion

The treatment of advanced metastatic CRCs remains a challenge; thus, exploring new target molecules and therapeutic strategies is of paramount importance. The research progress of Wnt/β-catenin signaling pathway mechanisms has accelerated the discovery of new therapeutic methods targeting Wnt/β-catenin pathway in CRCs. Although most drugs are still in the very early stages of research, it is expected that they will help in curing the intractable CRCs in the near future.

CAFs are critical ingredients of the TME with diverse effects, such as changing matrix deposition and remolding, intensive mutual signal interactions with cancer cells and interplay with infiltrating T cells (33). CAFs play a key role in cancer progression and metastasis; however, their prometastatic mechanisms have not been investigated. CAFs are abundant in CRC (34), but it is still unclear where they are recruited from, whether they may enhance metastasis-promoting communication, and how they exchange information with cancer cells and cross talk with the TME. CRC is the most common cancer in the world, and faces an enormous therapeutic challenge. Surgery, radiotherapy, and chemotherapy combined with targeted drugs have advanced the treatment for early-stage CRC. However, the main cause of death and metastasis remains poorly understood (35). Recent reports have demonstrated that Wnt signaling pathway activation induces primary resistance to immunotherapy, and CAFs were suggested to interact with Wnt signaling pathway (36).

We explored the CAFs to clarify how they remodel the TME through inhibition of the Wnt signaling pathway by hsBCL9_CT_-24 treatment or *Bcl9* knockdown. Pharmacological inhibition of Bcl9 in mice and genetic knockdown of *Bcl9* with shRNA in CT26 tumor cells was performed. We grouped the cells into types by using of graph-based clustering on the informative principal components. This technique identified cell clusters by marker genes, *S100a9*, *Pdgfrb*, and *Ptx3*, which were highly expressed in clusters of hsBCL9_CT_-24–treated group. In CT26 tumor cells group where *Bcl9* was knocked down by shRNA, *Rgs5*, *Dmkn*, and *Ass1* were highly expressed. *S100a9* and *Rg55* are marker genes of CAFs. We found that *Bcl9* depletion can cause protumor effect in CAFs.

Tumor stromal cells are necessary in tumorigenesis and tumor cells proliferation. Development and metastasis in the TME influences therapeutic response and clinical outcome, as well as interplay with peripheral cells (37) through the circulatory and immunologic systems to affect the development and progression of cancer (30). Moreover, the stroma cells of the TME such as CAFs play key roles in carcinogenesis through stimulating and facilitating uncontrolled cell proliferation. We explored the cell interaction between NT and KD groups. The TGFβ-TGFβR and EGFR-NRG1 binding between receptor and ligand was obvious in CAF/CT26 subgroups, revealing that the CAFs and CT26 tumor cells cross talk through TGF pathway and affect the expression of EGFR to transform the tumor cells. Therefore, stromal cells such as CAFs are a promising target for optimizing therapeutic strategies against cancer. These results illustrate that inhibition of BCL9 can reduce CAF communication with tumor cells.

GSVA analysis manifested immunologic signatures pathway such as TGF as the enriched signature in tumor cells. We applied SCENIC analysis to unravel which transcription factors determine differences in CT26 tumor cell expression between NT and *Bcl9* KD tumor cells. We also identified Foxp4 as a candidate transcription factor underlying gene expression differences in NT and *Bcl9* KD tumor cells. KEGG pathways analysis in CAF clusters was performed. For example, in CAF1, Wnt signaling and NF-κB signaling were enriched.

IL-2 is a pleiotropic cytokine, and control the differentiation and homeostasis of both pro- and anti-inflammatory T cells is fundamental to determining the molecular details of immune regulation. The IL-2 receptor couples to JAK tyrosine kinases and activates the STAT5 transcription factors. Our data revealed that IL-2 and STAT1 was enriched in tumor cells (**Figure 3D**). TGF-β has been shown to play an essential role in establishing immunological tolerance TGF-β inhibits the proliferation of T cells as well as cytokine production *via* Foxp3-dependent and independent mechanisms. Our data shows that the TGFβ-TGFβR and EGFR-NRG1 interaction revealing a cross-talk between the CAFs and CT26 tumor cells, which changes the expression of EGFR consequentially transforming the tumor cells (**Figures 5A, B** **)**. Bcl9 depletion could potentially benefit T-cell–mediated antitumor immune *via* modulating IL2 and TGFβ signaling.

In human colorectal tissue and tumor cells, a direct comparison of tumor cells between metastasis and non-metastasis tumors by GSVA analysis revealed that PTEN pathway, SMAD2 pathway, FGFR4 pathway, and JAK pathway were the enriched signatures in tumor cells. To assess which transcription factors underlie differences in tumor cell expression between metastasis and non-metastasis tumor cells SCENIC analysis was applied. We also identified STAT1 as a candidate transcription factor underlying gene expression differences in metastasis and non-metastasis tumor cells. STAT1 and FOS are associated with IFNγ signaling, which explains why the restraint of BCL9 can enhance the antitumor immune responses.

## Data Availability Statement

The data presented in the study are deposited in the FigShare repository, accession number/link: https://figshare.com/s/cd81407e68b809231131.

## Ethics Statement

The animal study was reviewed and approved by School of Pharmacy, Fudan University.

## Author Contributions

MY prepared the figures and wrote the manuscript. ZW analyzed the data. MF prepared the samples. YZ analyzed the data and edited the manuscript. YC performed analysis and edited manuscript. DZ conceptualized and wrote the manuscript. All authors contributed to the article and approved the submitted version.

## Funding

The current study was supported by the Science and Technology Commission of Shanghai (18ZR1403900 to DZ; and 18JC1413800 to DZ), the National Natural Science Foundation of China (81872895) (DZ), the project on joint translational research in Shanghai Institute of Materia Medica and Fudan University (FU-SIMM20181010) (DZ).

## Conflict of Interest

The author declares that the research was conducted in the absence of any commercial or financial relationships that could be construed as a potential conflict of interest.
