# Supplementary material for: Pharmacological Inhibition and Genetic Knockdown of BCL9 Modulate the Cellular Landscape of Cancer-Associated Fibroblasts in the Tumor-Immune Microenvironment of Colorectal Cancer
Source: Front Oncol. 2021 May 5;11:603556. doi: 10.3389/fonc.2021.603556 (PMC8131873; doi:10.3389/fonc.2021.603556)
Supplement: Supplementary file 1 [file DataSheet_1.docx]

Supplementary Material

# Supplementary Figures


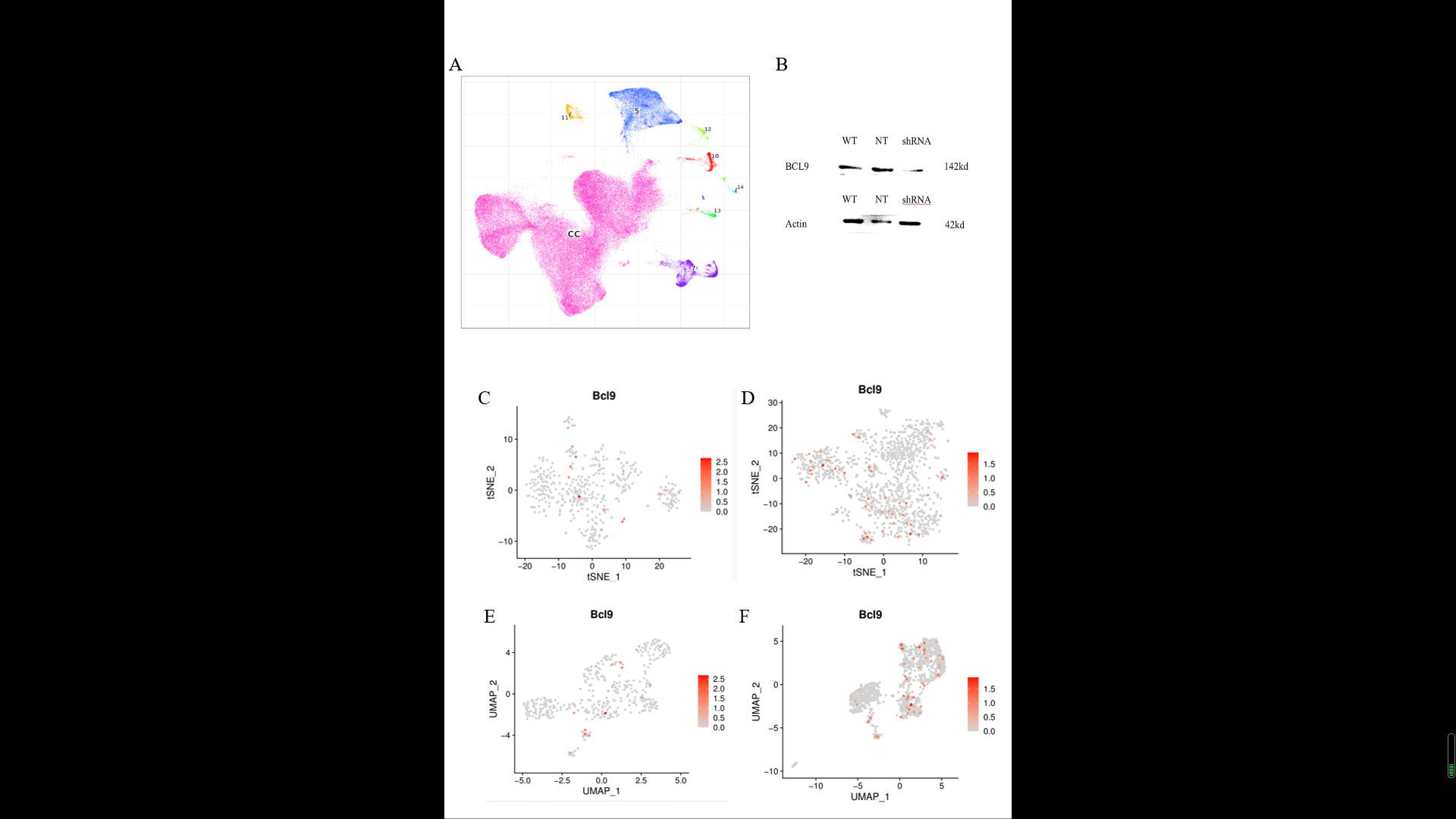


**Supplementary Figure 1. Single cell analysis of CT26 tumors.** (A) t-SNE of 12 tumor samples of CT26. Cluster CC: CT26 cancer cell; cluster 11: fibroblast; cluster 5: macrophage; cluster 7: T cell; cluster 10: granulocytes. (B) Western blot of Bcl9 and actin in wildtype (WT), Non-targeting (NT), Bcl9-shRNA transfected CT26 cells. (C) Bcl9 expression by TSNE of CT26 tumor treated with vehicle and hsBCL9_CT_-24. (D) Bcl9 expression by TSNE of CT26 tumor transfected with NT-shRNA and Bcl9-shRNA. (E) Bcl9 expression by UMAP of CT26 tumor treated with vehicle and hsBCL9_CT_-24. (F) Bcl9 expression by UMAP of CT26 tumor transfected with NT-shRNA and Bcl9-shRNA.


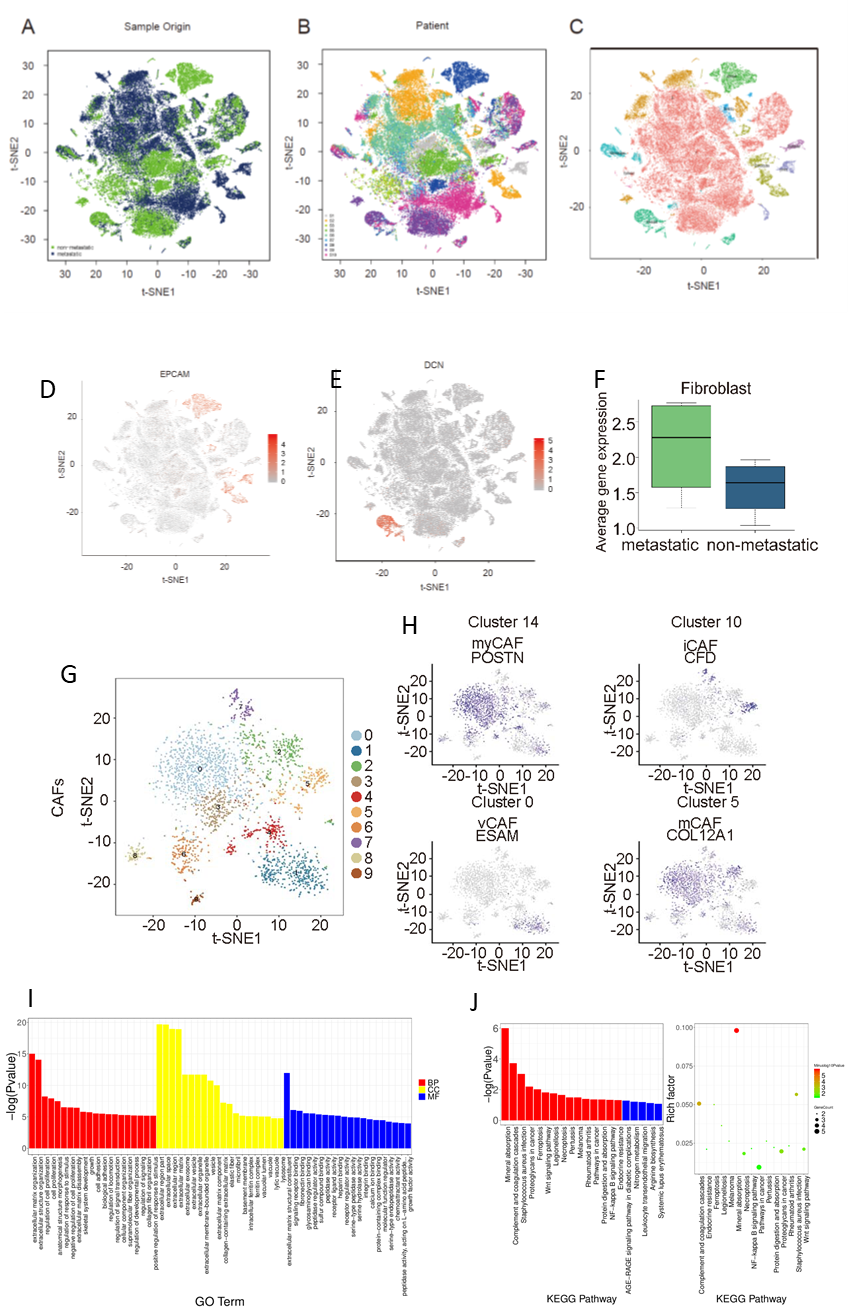


**Supplementary Figure 2**. Human CAFs cell analysis. (A) tSNE plot of 2549 CAFs, tSNE of the corresponding patients (non-metastatic or RCC-liver metastatic). (B) The associated 7 cell types of CAFs. (C) The fraction of different clusters of CAFs originating from non-metastatic and metastatic samples (left), the fraction of different clusters of CAFs originating from the 6 patients (middle), and the number of cells in different clusters (right). (D) Expression of S100A4 in CAFs. (E) Expression of VIM in CAFs. (F) Cell fraction of metastasis and non-metastasis of fibroblast. (G) tSNE plot of fibroblast. (H) Expression of POSTN, CFD, ESAM and COL12A1 in CAFs. tSNE plot, expression of marker gene for the 4 identified CAFs types defined above each panel. (I-J) GO and KEGG analysis of CAFs. Data were analyzed using a 2-sided t-test. ‘*’ means p<0.05.


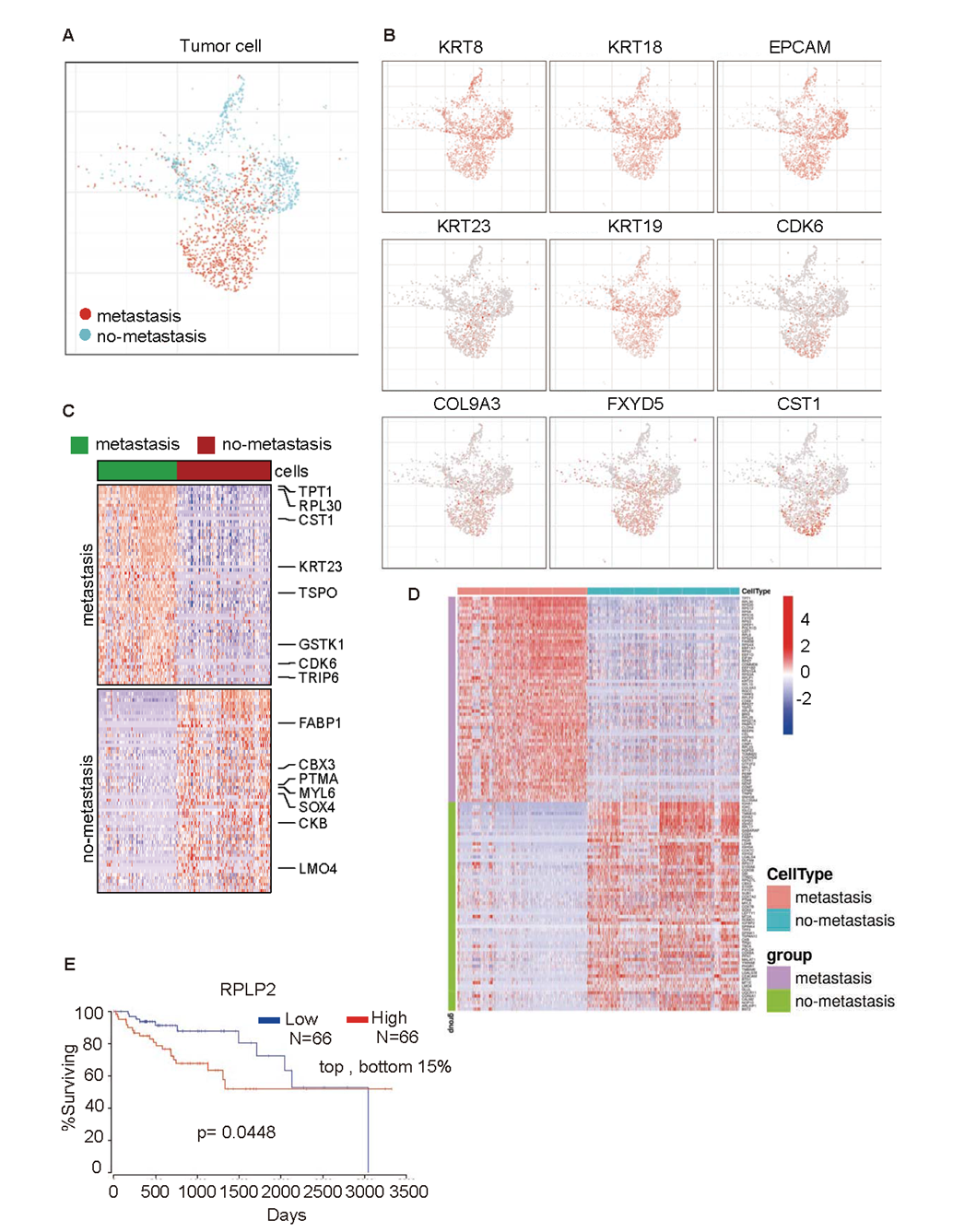


**Supplementary Figure 3**: Human tumor cells analysis. (A) tSNE plot of tumor cells. Its sample type of origin (non-metastatic or metastatic). (B) tSNE plot, expression of marker gene for the 9 different markers of tumor cell defined above each panel. (C-D) Differences in pathway activities analyzed by SCENIC between metastatic and non-metastatic T-cells. (E) Marker gene expression for tumor cells. Kaplan–Meier disease free survival curves for patients with CRC in TCGA. Stratification for high expression of RPLP2 versus low expression of RPLP2 was employed.


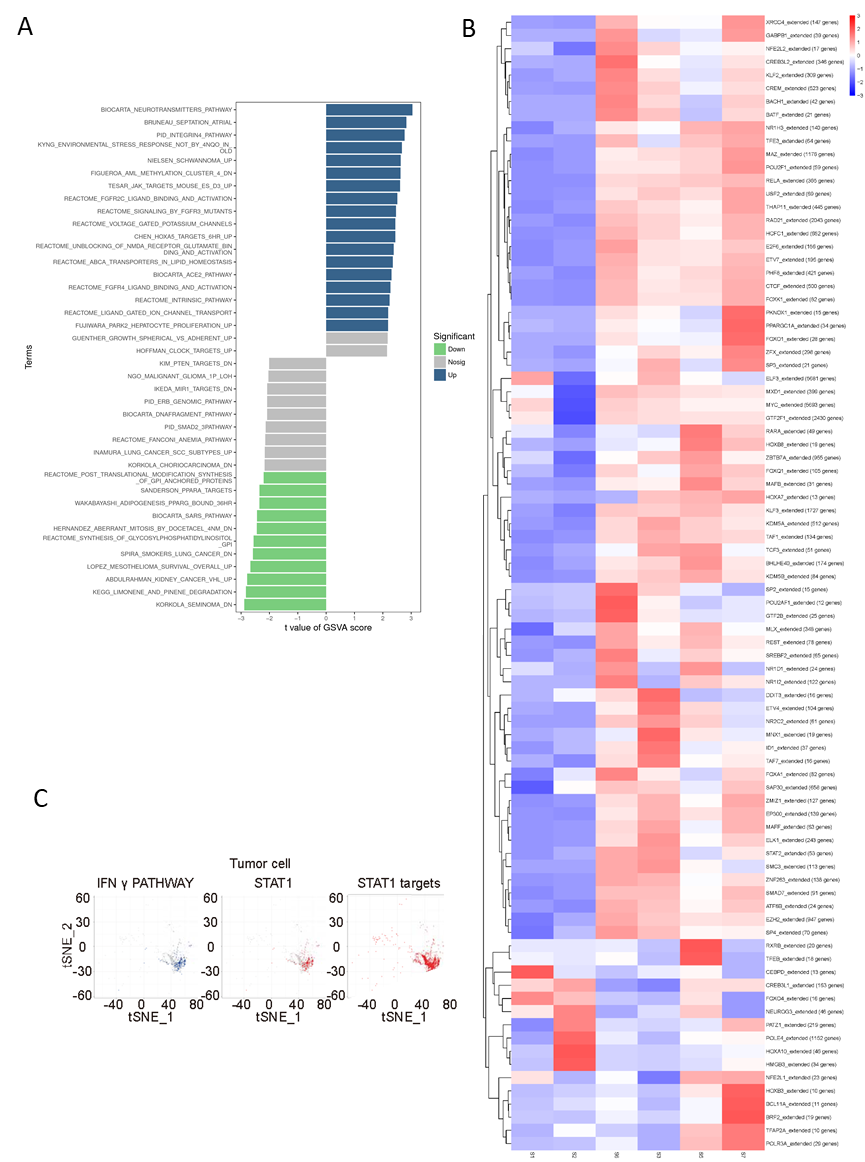


**Supplementary Figure 4.** GVAS and SCINIC analysis of human tumor cell. (A) Differences in pathway activities in CAF analyzed by GSVA between non-metastasis and metastasis. (B) Differences in transcription factors in CAF analyzed by SCENIC between non-metastasis and metastasis. (C) STAT1 and its associated pathways in tumor cells analyzed by SCENIC.

**Supplementary Table1**. Detailed information on CT26 mouse tumor specimens

| **ID** | **Animal Age** | **Animal Sex** | **Tumor Location** | **Treatment** | **Size(mm)** |
| --- | --- | --- | --- | --- | --- |
| 5 | 10 weeks | Female | s.c. | Vehicle | 13*10*5 |
| 8 | 10 weeks | Female | s.c | Vehicle | 12.2*9*4.5 |
| 10 | 10 weeks | Female | s.c | Vehicle | 13.4*8.6*4.3 |
| 4 | 10 weeks | Female | s.c | hsBCL9_CT_-24 | 14.3*4.4*2.2 |
| 12 | 10 weeks | Female | s.c | hsBCL9_CT_-24 | 11.1*4.6*2.3 |
| 16 | 10 weeks | Female | s.c | hsBCL9_CT_-24 | 13.03*6.4*3.2 |
| 18 | 10 weeks | Female | s.c | NT-shRNA | 16.5*8.4*4.2 |
| 19 | 10 weeks | Female | s.c | NT-shRNA | 12.3*10*5 |
| 22 | 10 weeks | Female | s.c | NT-shRNA | 11*10*5 |
| 24 | 10 weeks | Female | s.c | Bcl9-shRNA | 10*7.2*3.6 |
| 25 | 10 weeks | Female | s.c | Bcl9-shRNA | 9*7.4*3.7 |
| 29 | 10 weeks | Female | s.c | Bcl9-shRNA | 8.4*7.6*3.8 |
|  |  |  |  |  |  |
